# A Case Report of X-Linked Hyperimmunoglobulin M Syndrome with Lipoma Arborescens of Knees

**DOI:** 10.1155/2016/5797232

**Published:** 2016-10-12

**Authors:** Qiuting Dong, Jinxia Zhao, Zhongqiang Yao, Xiangyuan Liu, Huiying He

**Affiliations:** ^1^Department of Rheumatology & Immunology, Peking University Third Hospital, 49 North Garden Rd., Haidian District, Beijing 100191, China; ^2^Center for Coronary Heart Disease, Fu Wai Hospital and Cardiovascular Institute, Peking Union Medical College and Chinese Academy of Medical Sciences, 167 Beilishi Rd., Beijing 100037, China; ^3^Department of Pathology, Peking University Third Hospital, 49 North Garden Rd., Haidian District, Beijing 100191, China

## Abstract

The X-linked hyperimmunoglobulin M syndrome (HIGM), caused by mutations in the CD40LG gene, is a kind of primary immunodeficiency disease (PID). Patients with X-linked HIGM are susceptible to infection as well as autoimmune diseases. Lipoma arborescens (LA) is a rare benign tumor, of which the pathogenesis mechanism has not been clearly understood. We report a case of HIGM combined with LA in a 22-year-old male patient. A new deletion mutation of CD40LG gene was detected in this case. The possible relationship between HIGM and LA was also discussed.

## 1. Introduction

The hyperimmunoglobulin M syndrome (HIGM) is a heterogeneous group of primary immunodeficiency disease (PID) caused by the defects of immunoglobulin class switch recombination which lead to the deficiency of IgG, IgA, and IgE with preserved or elevated levels of IgM [1]. Patients with HIGM are susceptible to recurrent and opportunistic infections. These patients are also prone to autoimmune disorders, such as arthritis, hematologic abnormalities, and inflammatory bowel disease [2]. Inflammatory arthritis has been reported in male patients with X-linked HIGM [3] and in patients with an autosomal recessive form of HIGM [4].

Synovial lipoma arborescens (LA) is a rare intra-articular benign tumor, characterized by the diffuse substitution of the synovial tissue by mature adipocytes, resulting in a villous lipomatous proliferation [5]. LA is most commonly seen in the knee and usually occurs as painless swelling with intermittent exacerbations. The pathogenesis of LA has not been clearly elucidated. There is no literature describing the relationship between HIGM and LA by now. We presented a case of HIGM combined with LA in a 22-year-old male patient and discussed the possible correlation between HIGM and LA.

## 2. Case Report

A 22-year-old male patient complained about a history of painless recurrent swelling of both knees for 17 years. He was susceptible to upper respiratory tract infections since 3 years old. He was diagnosed as having aseptic synovitis at 5 years old and treated with prednisolone for 3 months. However, the joint swelling progressively aggravated. Six years ago, he received arthroscopic synovectomy with complete resolution of the clinical symptoms. The pathology of synovial biopsy showed chronic hyperplasia synovitis with fatty infiltration. Four years ago, the symptom of joint swelling relapsed. The painless swelling of both knees was persistent, without deformity of joints. He then came to our hospital for further diagnosis and treatment.

**Figure 1:**
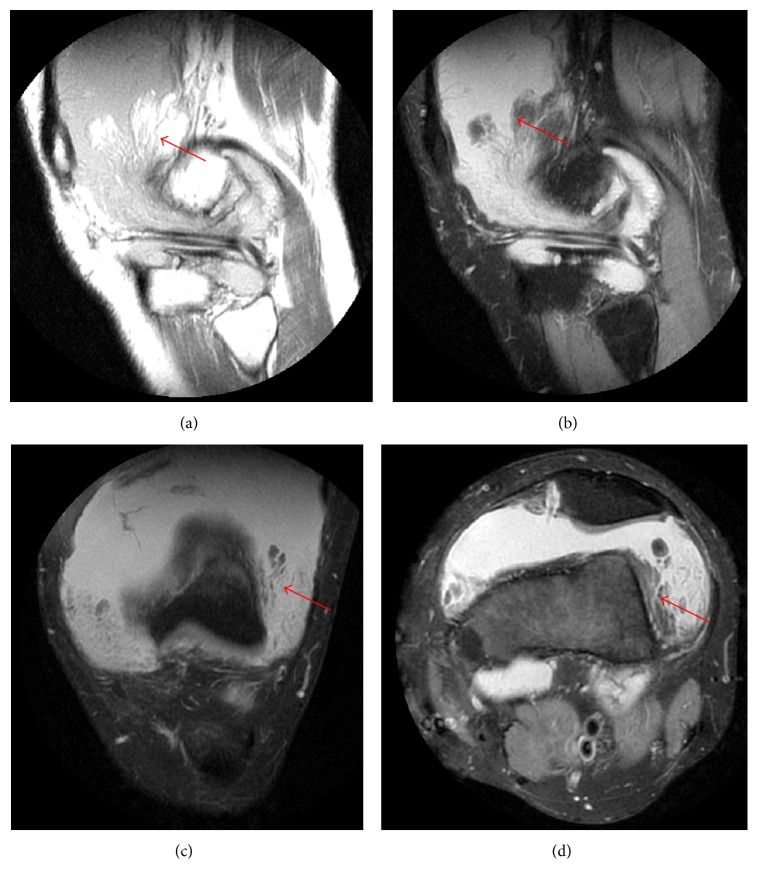
MRI of a 22-year-old male patient with lipoma arborescens of knee joints. Sagittal noncontrast T1 (b), weighted image, showed high signal intensity frond like synovial thickening in the suprapatellar bursal region (arrow) with joint effusion. GE HDesigna 3.0 Tesla magnetic resonance imaging system TR-737ms TE-9.3ms 3.5 mm slice thickness with interslice gap of 1 mm was used. Sagittal (a), coronal (c), and axial (d) fat suppressed proton density images revealed complete suppression of signal intensity of villous projections (arrow) in suprapatellar bursal region. GE HDesigna 3.0 Tesla magnetic resonance imaging system was used. Sagittal TR-2264ms TE-24ms 3.5 mm slice thickness with interslice gap of 1 mm, coronal TR-2740 TE-24 3.5 mm slice thickness with interslice gap of 0.5 mm, and axial TR-1956 TE-22.8 4 mm slice thickness with interslice gap of 1 mm.

**Figure 2:**
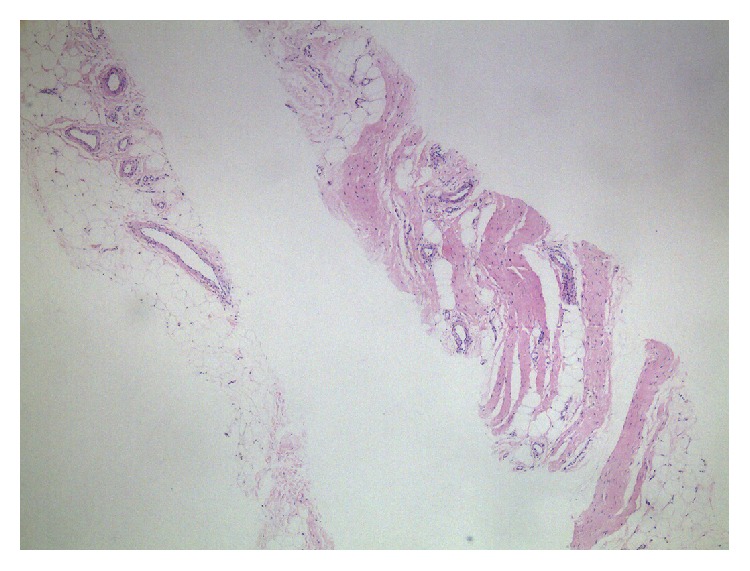
Histopathologic changes of synovial biopsy. Histopathologic examination of synovial biopsy revealed multiple villi having fatty core lined by hyperplastic synoviocytes with chronic inflammatory cell infiltration (hematoxylin and eosin stain 4x).

The physical examination revealed that both knees were swelling without tenderness. Fluctuation and patellar tap test was positive. The magnetic resonance imaging (MRI) of the knees showed massive effusion associated with multiple frond like synovial thickening of high signal intensity in sagittal T1 weighted image especially in the suprapatellar region (Figure 1). Fat suppressed proton density images (Figure 1) revealed complete suppression of signal intensity of villous projections in suprapatellar region. Synovial biopsy guided by the ultrasound was performed and the histopathologic examination revealed a fibroadipose synovial membrane with small vascular proliferation and a small amount of lymphocytes and mononuclear cells infiltration (Figure 2). The diagnosis of LA was made according to the features of MRI and synovial pathology.

**Figure 3:**
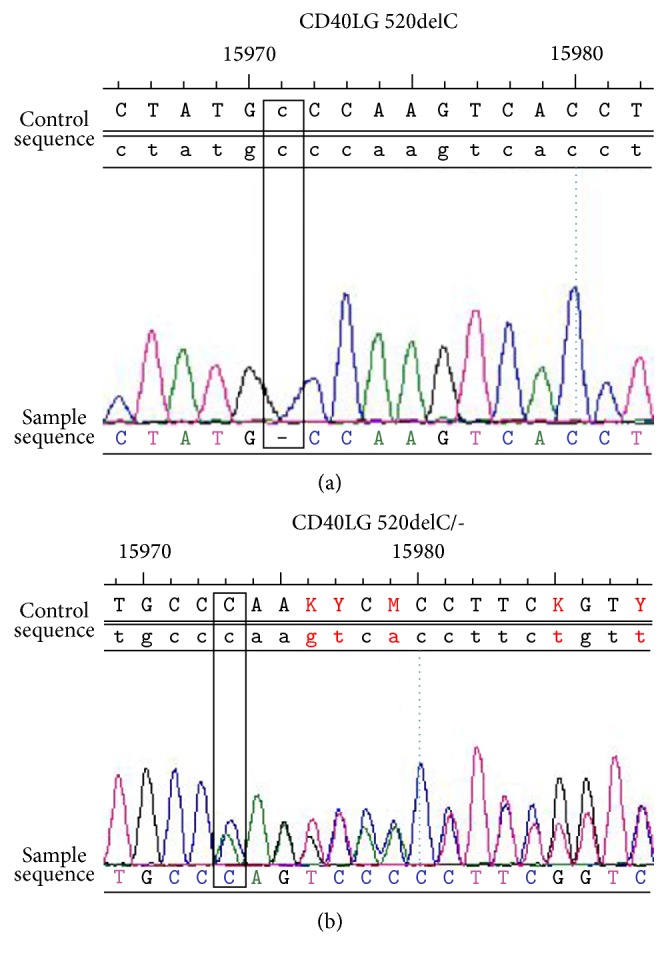
CD40LG gene analysis of the patient and his mother. CD40LG gene analysis showed the deletion mutation of cytidylic acid residue at position 520 in exon 5 region in the patient (a). The patient's mother carried the same mutation (b).

The laboratory tests including autoantibodies, HIV, HBsAg, HCV-Ab, and syphilis antibody were normal. Cytologic and biochemical examinations of synovial fluid were normal. Synovial fluid culture showed no growth of pathogen. The lymphocyte culture and interferon determination and synovial fluid analysis for acid-fast bacilli were negative which excluded the tuberculosis infection. There were no history or clinical and pathological findings which were suggestive of infectious arthritis. However, the ESR was 90 mm/h (0–15 mm/h) and CRP was 1.3 mg/dL (0–0.8 mg/dL). The blood routine showed neutropenia and immunoglobulin test revealed deficiency of IgG (0.34 g/L, normal range 6.94–16.18 g/L) and IgA (0.07 g/L, normal range 0.7–3.8 g/L) with high level of IgM (23 g/L, normal range 0.6–2.3 g/L). Bone marrow examination was normal. He was suspected to have hyperimmunoglobulin M syndrome (HIGM). Gene test was performed to confirm the type of HIGM. It revealed a deletion of cytidylic acid residue in exon 5 region at position 520 (c.520delC) which led to a shift in the encoding of the reading frame from position 174 (p.Gln174fs). Premature termination occurred in 190 amino acids (numbering according to Hollenbaugh et al. [6]; Figure 3(a)). The patient's mother carried the same mutation (Figure 3(b)). Therefore, the diagnosis of X-linked HIMG was made. Informed consent for sequence analysis was obtained from the patient and his mother.

Interestingly, splenic hemangioma (5.8*∗*5.0 cm) was found by the contrast abdominal CT scan. He had no symptoms. There was no surgical indication of the vascular tumor. We suggested he follow up closely with regular ultrasound examination once a year.

The patients received synovectomy operation and were given regular IVIG therapy with 15 g *∗* 3 days (total dose of 600 mg/kg, 75 kg) every month. No relapse of LA or infection occurred during one-year follow-up period.

## 3. Discussion

We report a case of X-linked HIGM with a novel mutation. The X-linked HIGM, caused by mutations in the CD40LG gene (previously known as TNFSF5 or CD154), is a kind of PID with the characteristics of immunoglobulin class switch defect leading to deficiency of IgG, IgA, and IgE. Mutations in the CD40LG gene also impair the T cells' ability to differentiate and interact with immune system cells [7]. Therefore, patients with X-linked HIGM are more susceptible to infections.

Although patients with X-linked HIGM usually have the complications of infections and autoimmune diseases, there is no literature revealing the relationship between X-linked HIGM and LA. LA is a chronic, progressive intra-articular benign tumor usually involving the knee joint characterized by villous lipomatous proliferation of the synovium. The diagnosis of LA is made mainly by the imaging and pathologic features. It is reported that LA could be observed in patients aged between 9 and 68 years, with equal predominance in men and women [8, 9]. The etiology of LA has not been clearly explained. Most secondary LA is caused by chronic irritation, such as degenerative disease, trauma, meniscal injury, or synovitis, and occurs in older patients [10]. The primary LA is idiopathic and occurs at a younger age. In our case report, the patient showed symptom of joint swelling at the age of 5 years old. He did not take the MRI or biopsy until 16 years old. Although the LA and X-linked HIGM may be two separate diseases, the immunodeficiency disease may have contributed to the development of LA in this case.

The treatment of X-linked HIGM is bone marrow transplantation or immunoglobulin replacement therapy. The treatment of LA is usually surgery when conservative management failed. The surgical treatment of LA could be either open or arthroscopic synovectomy. In this case report, the patient received the arthroscopic synovectomy 6 years ago. However, the LA reoccurred after 2 years of the operation. This indicated that LA could be recurrent if the first operation did not clean the lesions completely.

## 4. Conclusion

We reported a CD40L gene deficient X-linked HIGM patient complicated with bilateral LA of the knee and splenic hemangioma. A new mutation of CD40L gene was found in this patient. The relationship between the occurrence of LA and HIGM remains to be illustrated.
